# Can Timely Vector Control Interventions Triggered by Atypical Environmental Conditions Prevent Malaria Epidemics? A Case-Study from Wajir County, Kenya

**DOI:** 10.1371/journal.pone.0092386

**Published:** 2014-04-03

**Authors:** Peter Maes, Anthony D. Harries, Rafael Van den Bergh, Abdisalan Noor, Robert W. Snow, Katherine Tayler-Smith, Sven Gudmund Hinderaker, Rony Zachariah, Richard Allan

**Affiliations:** 1 Medical Department, Water, Hygiene and Sanitation Unit, Médecins Sans Frontières, Operational Center Brussels, Brussels, Belgium; 2 International Union Against Tuberculosis and Lung Disease (The Union), Paris, France; 3 London School of Hygiene and Tropical Medicine, London, United Kingdom; 4 Medical Department, Operational Research Unit (LuxOR), Operational Center Brussels, Médecins Sans Frontières -Luxembourg, Luxembourg, Luxembourg; 5 Malaria Public Health Department, KEMRI-University of Oxford-Wellcome Trust Collaborative Programme, Nairobi, Kenya; 6 Centre for Tropical Medicine, University of Oxford, Oxford, United Kingdom; 7 Centre for International Health, University of Bergen, Bergen, Norway; 8 The Mentor Initiative, Crawley, United Kingdom; Université Pierre et Marie Curie, France

## Abstract

**Background:**

Atypical environmental conditions with drought followed by heavy rainfall and flooding in arid areas in sub-Saharan Africa can lead to explosive epidemics of malaria, which might be prevented through timely vector-control interventions.

**Objectives:**

Wajir County in Northeast Kenya is classified as having seasonal malaria transmission. The aim of this study was to describe in Wajir town the environmental conditions, the scope and timing of vector-control interventions and the associated resulting burden of malaria at two time periods (1996–1998 and 2005–2007).

**Methods:**

This is a cross-sectional descriptive and ecological study using data collected for routine program monitoring and evaluation.

**Results:**

In both time periods, there were atypical environmental conditions with drought and malnutrition followed by massive monthly rainfall resulting in flooding and animal/human Rift Valley Fever. In 1998, this was associated with a large and explosive malaria epidemic (weekly incidence rates peaking at 54/1,000 population/week) with vector-control interventions starting over six months after the massive rainfall and when the malaria epidemic was abating. In 2007, vector-control interventions started sooner within about three months after the massive rainfall and no malaria epidemic was recorded with weekly malaria incidence rates never exceeding 0.5 per 1,000 population per week.

**Discussion and Conclusion:**

Did timely vector-control interventions in Wajir town prevent a malaria epidemic? In 2007, the neighboring county of Garissa experienced similar climatic events as Wajir, but vector-control interventions started six months after the heavy un-seasonal rainfall and large scale flooding resulted in a malaria epidemic with monthly incidence rates peaking at 40/1,000 population. In conclusion, this study suggests that atypical environmental conditions can herald a malaria outbreak in certain settings. In turn, this should alert responsible stakeholders about the need to act rapidly and preemptively with appropriate and wide-scale vector-control interventions to mitigate the risk.

## Introduction

According to the World Health Organization (WHO) 2011 malaria report, there were 216 million cases of malaria globally in 2010, with over 80% occurring in sub-Saharan Africa [1]. There were 655,000 deaths, with 86% of the victims being children under the age of five years and 91% of malaria deaths occurring in Africa.

In Kenya, like many other African countries, malaria is a leading cause of morbidity and mortality, especially amongst children. Kenya has four malaria epidemiological zones: an endemic zone; seasonal malaria transmission zones; zones that are prone to malaria epidemics, and a zone of low malaria risk. There are limited data on effectiveness of interventions to prevent or control malaria outbreaks in zones where seasonal transmission or epidemics of malaria may occur. Control measures, when taken, are often implemented too late and with minimal coordination and expertise, and often under intense political pressure [2].

Wajir County in Northeast Kenya is usually dry and hot and is a zone classified as having seasonal malaria transmission. When certain environmental conditions occur, epidemics of malaria may ensue. Since 1932, there have been two recorded major malaria epidemics in the county, one in 1961 and one in late 1997. The epidemic in 1997–1998 was preceded by twelve months of high temperatures and drought that led to widespread malnutrition [3]. El Niño then caused virtually uninterrupted rainfall and the worst flooding in the county for over 50 years, resulting in large scale population displacement, and an outbreak of Rift Valley Fever (RVF) in the county. The remaining shallow flood waters covered an extensive area, and provided ideal breeding conditions for *Anopheles* mosquitoes (malaria vectors). This resulted in an exponential growth of the vector population and an explosive epidemic of malaria [4]. The poor nutritional status of the population, displacement from their homes, and lack of access to treatment services due to flooding and a national health staff strike, made individuals highly susceptible to malaria infection and to developing severe disease. Between February and May 1998, a total of 23,377 malaria cases (a malaria attack rate of 39% in the population) were reported [5]. The average crude mortality was approximately 9 per 10,000 per day and in the under-5 population this rose to approximately 28 per 10,000 per day.

Similar environmental conditions occurred in 2007 with protracted drought resulting in wide-spread under-nutrition, followed by very heavy rainfall and flooding across much of the province, causing mass population displacement and a RVF outbreak in the county. Prompted by these environmental conditions and before malaria cases had increased above normal baseline numbers, Médecins Sans Frontières (MSF) intervened with an emergency approach designed to mitigate the risk of a malaria epidemic occurring. The ongoing surveillance and case management activities in the county were promptly reinforced. Also, within ten days, malaria vector control interventions were launched with the aim of cutting malaria transmission and mitigating against the risk of a large scale malaria epidemic developing, as had occurred in 1997.

The aim of this study is to describe certain environmental conditions and the associated resulting burden of malaria in Wajir town at two time periods (1996–1998 and 2005–2007), each characterized by vector control interventions which started at different phases of the malaria transmission process.

## Methods

### Study Design, Data Variables and Sources

This was a cross-sectional descriptive and ecological study using previously collected data.

### Data variables

The following data variables were collected:

i) for environmental conditions and their impact on the population: -daily temperature (°C); rainfall (mm per month); reported flooding in the county (yes/no); internationally reported cases of animal and/or human RVF in the county (yes/no); and malnutrition and severe acute malnutrition in the under-fives in the county and Wajir town as measured by two stage cluster surveys and defined by z-scores [6]: ii) for malaria vector control interventions:- number of houses that received indoor residual spraying; number of long-lasting impregnated nets (LLIN) distributed; and number of surface flood water bodies where larvicides were applied: iii) for malaria:- number of admissions each month in Wajir hospital diagnosed with malaria based on clinical features; number of persons diagnosed with malaria each week in Wajir town per 1000 population (weekly malaria incidence). For the weekly malaria incidence figures, malaria was diagnosed based on clinical features of “any patient reporting a recent history of fever, in the absence of any other cause” confirmed by thin blood smears or malaria rapid diagnostic tests (Paracheck-Pf).

### Data sources

Sources of data for the study included:- i) A Wellcome Trust electronic Excel 2003 database on rainfall, temperature and malaria from 1999 to 2008 [7]; ii) A Kenya Meteorological Department, Wajir station Excel 2003 data base for temperature and rainfall from 1998 to 2006; iii) MSF-Epicentre internal electronic reports on flooding, malnutrition and RVF cases in 1997/1998 and 2007; iv) MSF-Epicentre internal electronic reports on malaria vector control interventions in 1998 and 2007; v) MSF Mobile Clinic registers and Ministry of Health hospital and county reports on malaria cases in 1997/1998 and 2007; vi) The MENTOR Initiative internal electronic reports on a 2007 emergency Malaria Control intervention for the most vulnerable flood affected communities in Kenya; and vii) The Nutrition Information in Crisis Situation electronic database by the United Nations system Standing Committee on Nutrition for nutritional information..

### Setting

#### General

Kenya is a large country in East Africa with a population of 42 million in 2011, according to the World Bank. It has eight provinces including the North Eastern Province, which was, at the time, divided into four districts, now counties, from north to south; Mandera, Wajir, Garissa and Ijara.

#### Wajir County

The study took place in Wajir County which is the second largest county in Kenya, covering an area of 56,501 km^2^ [3]. Wajir County ranges from very arid in the north, to semi-arid in the south. In 2007, the county had 13 administrative divisions with a total of 74 locations and 88 sub-locations. Wajir County includes Wajir town, which consists of the centre of the town or Township surrounded by the five major villages of Jog Baru, Wagberi, Hodhan, Alimao and Barwaqo. The population was about 500,000 people with an annual growth of 2.5%, and the majority of the population consisted of nomadic pastoralists. Over 63% of the population depended solely on livestock for their livelihood, 57% lived in absolute poverty (<1 USD/day) and the overall literacy level was 12.5%. [8]. In 2007, the county reportedly had one district hospital in Wajir town, five sub-district hospitals, one health center and more than 30 dispensaries.

The county experiences the Sahel climate characterized by long dry spells and two rainy seasons each year from September to January and March to May. The mean annual precipitation between 1932 and 1998 was just over 300 mm [3]. These desert fringe conditions create seasonal malaria transmission during, and immediately following, the two rainy seasons. The intensity of the resulting malaria seasonal transmission varies considerably from year to year, depending largely on the climatic conditions. Temperatures are usually high and average rainfall creates surface water pools suitable for vector breeding sites. Extreme climatic conditions with unseasonal heavy rainfall can result in flooding in many parts of this county. Surface flood water, whilst initially washing away most existing mosquito larvae, creates unusually large vector breeding sites as the flood waters gradually recede and become shallow surface water in the following weeks. Mass vector breeding results in very high rates of malaria transmission, and epidemic outbreaks of malaria with high morbidity and mortality rates [9], [10], [11].

A RVF outbreak also requires heavy rainfall and extensive flooding of low lying grassland depressions associated with the rapid and mass emergence of *Aedes* mosquitoes reaching their maximum population about ten days after the flood event [12], [13], [14]. The breeding sites of the *Anopheles* mosquitoes that transmit malaria take much longer to recover from flooding compared with those of *Aedes*, and thus the *Anopheles* mosquito population reaches a maximum about one to two months after the flood event [15]. Thus, episodes of RVF often herald malaria epidemics in this sort of context.

#### Malaria control

In 1997/1998 MSF carried out water rehabilitation and cholera preparedness projects, and monitoring of RVF. MSF supported Wajir town hospital during this period, with out-patient and in-patient case management of malaria. Following atypical environmental conditions, an explosive malaria outbreak occurred in Wajir County between February and May 1998. MSF also started an Indoor Residual Spraying campaign in Wajir town about four to five weeks after the peak of the malaria outbreak in close collaboration with the District Public Health Office (DPHO). MERLIN, a UK non-governmental organization, worked in parallel with MSF, providing primary health care centre support, mobile clinics and insecticide treated bed-nets in flood affected areas in the county, but not in Wajir town. The Catholic mission, located in Wajir town provided additional clinical services, and the Swedish Rotarians had two volunteers providing laboratory and clinical support at the hospital. MSF left Wajir in 1998.

Heavy rains and flooding affected Wajir County and Greater Garissa from the end of September 2006, creating conditions very similar to those leading to the large malaria epidemic in 1997/1998. In response, MSF managed an outbreak of RVF in the neighbouring Garissa county and in January 2007 launched integrated malaria vector control activities in Wajir town.

### Study participants

Study participants included all adult and pediatric (less than 15 years of age) inpatients diagnosed with malaria at Wajir district hospital and all outpatients diagnosed with malaria in Wajir town during the two time periods (1996–1998 and 2005–2007).

### Analysis and statistics

Data were entered into an electronic Excel 2003 database and a simple descriptive analysis was carried out.

### Ethics statement

This study met the Médecins Sans Frontières' Ethics Review Board (Geneva, Switzerland) -approved criteria for analysis of routinely-collected program data, and was also approved by the Ethics Advisory Group of the International Union Against Tuberculosis and Lung Disease, Paris, France. Analysis was done on previously collected program data only: patients were not contacted, and no identifying characteristics of patients were collected. As such, informed consent of involved patients was not indicated or sought

## Results

### Environmental conditions and hospital malaria admissions: 1996–1998

**Figure 1:**
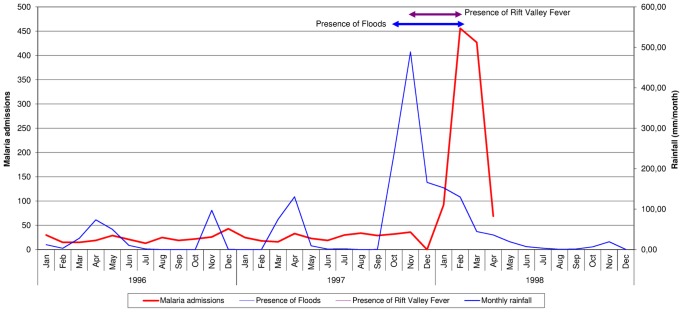
Monthly rainfall, presence of floods and Rift Valley Fever and malaria admissions to Wajir Hospital: January 1996–December 1998.

The monthly rainfall, the presence of floods and RVF and the number of malaria admissions to Wajir hospital between January 1996 and December 1998 are shown in **Figure 1**. The seasonal periods of rainfall followed by drought are shown up to August 1997. From September 1997 to February 1998 there was very heavy monthly rainfall, at its peak reaching nearly 500 mm for the month of November. This resulted in extensive flooding between the months of October 1997 to February 1998 and RVF from November 1997 to February 1998. The number of malaria admissions from January 1996 to December 1997 was relatively stable ranging from 13 to 43 per month (with the exception of December when there was a hospital strike and no admissions). The increase in malaria admissions was first noted in January 1998 with 92 cases for the month and this peaked at 456 in February, before dropping to 427 in March and 69 in April.

During this three year period the daily temperature never dropped below 21°C. The prevalence of malnutrition and severe acute malnutrition in children under the age of five years in the county and the town during the three year period is shown in **Table 1**.

**Table 1:** Documented prevalence of malnutrition in Wajir county and Wajir town in children under the age of five years from 1996–1998.

Site	Year Month	Prevalence of Malnutrition %	Prevalence of severe acute malnutrition %	Source of data
Wajir County	Oct 1996	27.9	3.8	OXFAM
	Feb 1997	25.1	4.2	MERLIN
	Jul 1997	8.8	1.0	OXFAM/MOH
	Mar 1998	16.0	2.9	OXFAM
Wajir Town	Jul 1997	15.0	1.7	OXFAM/MOH^1^
	Mar 1998	25.3	3.7	MSF

1 Ministry of Health.

### Environmental conditions and hospital malaria admissions: 2005–2007

**Figure 2:**
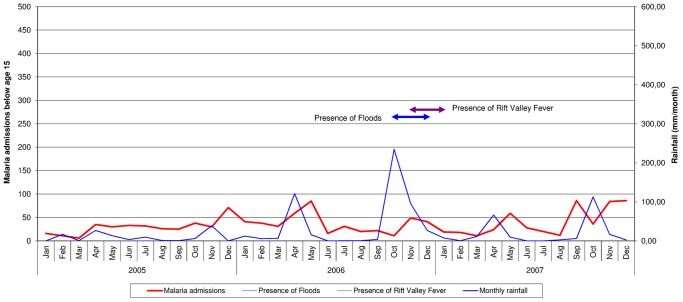
Monthly rainfall, presence of floods and Rift Valley Fever and malaria admissions in persons under 15 years of age to Wajir Hospital: January 2005–December 2007.

The monthly rainfall, the presence of floods and RVF, and the number of malaria admissions in persons aged 15 years and below to Wajir hospital from January 2005 to December 2007 are shown in **Figure 2**. The seasonal periods of rainfall followed by drought are shown up to September 2006. At the end of September 2006, very heavy rains began, and in October the monthly rainfall reached almost 250 mm, declining to 95 mm in November and then back to the monthly average. This resulted in widespread flooding between the months of October and December 2006 and a RVF outbreak from November 2006 to January 2007. The number of malaria admissions in persons under the age of 15 years remained relatively stable during the whole three year period and there was no increase in cases during or after the time of rainfall and flooding.

During this three year period the daily recorded temperature never fell below 22°C. The prevalence of malnutrition and severe acute malnutrition in children under the age of five years in the county was 15.6% and 4.1% respectively in May 2006 and 23.0% and 2.8% in April 2007.

### The scope of malaria vector control interventions: 1998 and 2007

The scope of malaria vector control interventions including the number of houses sprayed, LLINs distributed, modeled LLIN coverage based on the numbers distributed, and shallow pools treated with larvicides in Wajir town in 1998 and 2007 is shown in **Table 2**. Over 90% of houses were sprayed in the two periods, but in 1998 there were no LLINs distributed in the town, and no shallow pools were treated, in contrast to 2007.

**Table 2:** Scope of malaria control interventions in 1998 and 2007 in Wajir Town.

Malaria control interventions	1998	2007
Number of houses	9468	28536
Number (%) houses sprayed	9372 (99%)	26544 (93%)
Number of LLINs needed for 100% coverage	No data	45708
Number (coverage %) LLINs distributed	0	34716 (76%)
Shallow pools treated with larvicides	0	5

LLIN = long-lasting insecticide treated nets.

### Timing of malaria vector control interventions and malaria incidence: 1998 and 2007

**Figure 3:**
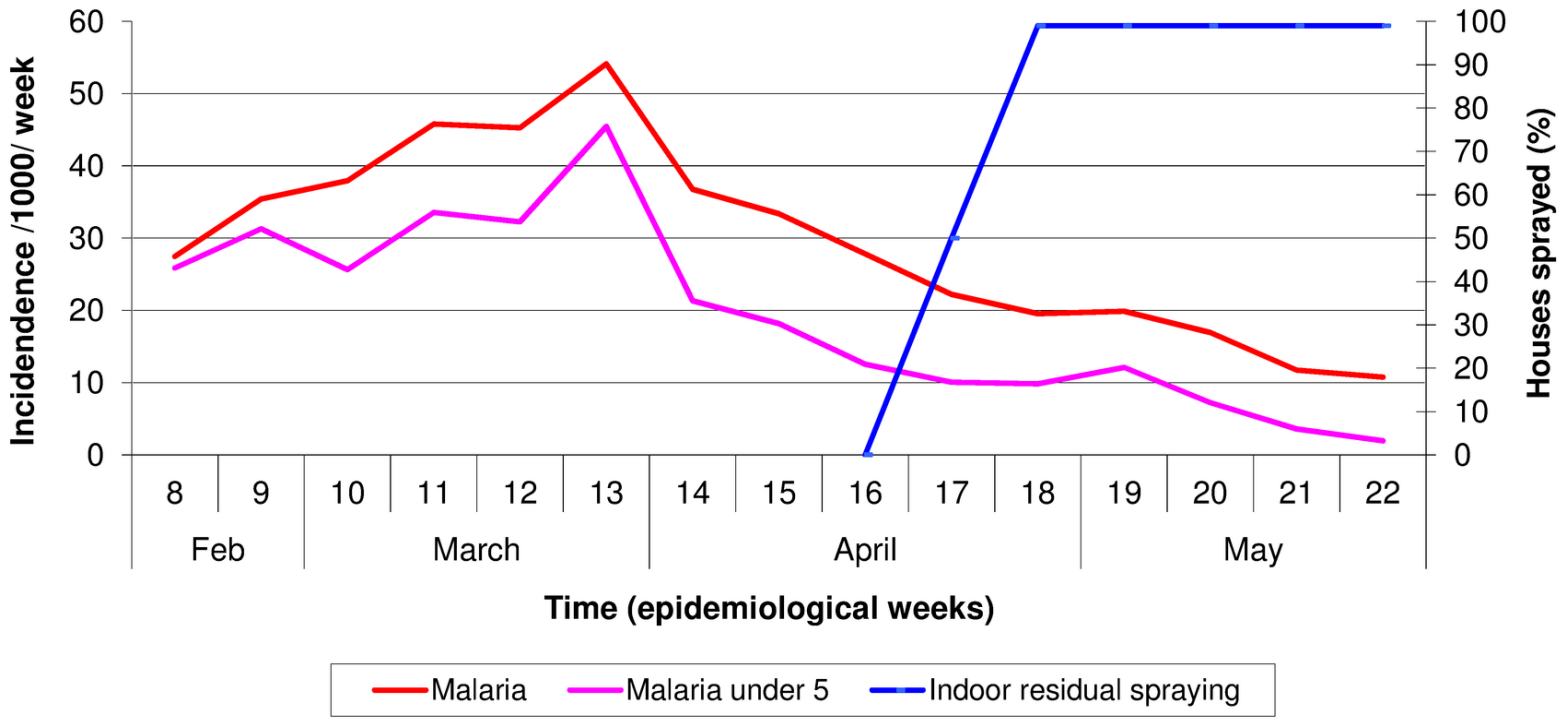
Incidence of malaria per 1000 population per week in Wajir town and timing of indoor residual spraying in 1998.

In 1998, the incidence of malaria per 1000 population per week and the timing of indoor residual spraying are shown in **Figure 3**. The incidence of all malaria cases and malaria in children under the age of five years peaked at the end of March 1998 at 54 per 1000 population per week and 46 per 1000 per week respectively. Indoor residual spraying started in mid-April reaching full house coverage of Wajir Town after two weeks. However, the start of this intervention occurred at a time when malaria incidence was already decreasing.

**Figure 4:**
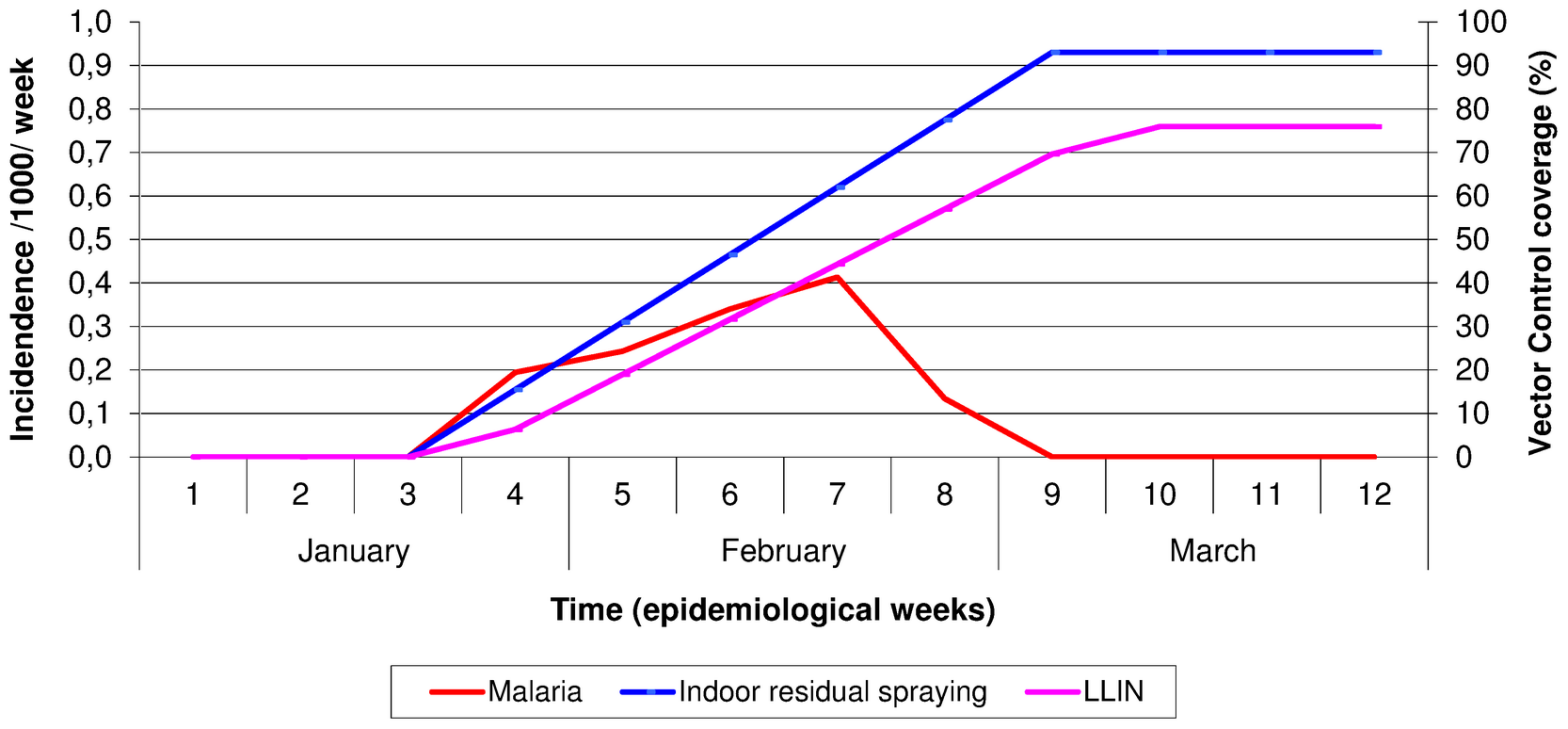
Incidence of malaria per 1000 population per week in Wajir town and timing of indoor residual spraying and modeled long-lasting insecticidal nets coverage in 2007.

In 2007, the incidence of malaria per 1000 population per week and the timing of indoor residual spraying and distribution of LLINs are shown in **Figure 4**. The two malaria vector control interventions illustrated in Figure 3 and the treatment of shallow pools with larvicides started in the third week of January before any increase in malaria incidence was observed. It took ten days between the decision to intervene and the start of activities. Seven weeks after starting activities, 93% of all houses in Wajir town were sprayed and there was 76% household coverage of LLINs meaning that 76% of Wajir town population had received one LLIN per 1.8 persons. The incidence of all malaria cases and malaria in children under the age of five years remained low at less than 0.5 per 1000 population per week between the months of January to March in Wajir Town. The malaria surveillance in the peripheral areas illustrated that the malaria incidence in Wajir County did not exceed 1 per 1000 population per week between the months of January to March.

## Discussion

This cross-sectional descriptive and ecological study showed that in 1997/1998 and 2006/2007 Wajir County in north-eastern Kenya experienced atypical environmental conditions with drought and malnutrition, followed by massive monthly rainfall resulting in flooding and animal/human RVF. In 1998, this was associated with a large and explosive epidemic of malaria resulting in large numbers of admissions to Wajir Hospital and a weekly malaria incidence of 40–55 cases per 1000 population per week in all persons and children. Vector control interventions at that time consisted solely of indoor residual spraying of houses, and these started over 6 months after the onset of heavy rains when the malaria epidemic was already abating. In 2007, as a result of similar environmental conditions, MSF instituted a package of vector control interventions that included indoor residual spraying, distribution of LLINs and applying larvicides to shallow pools. These interventions started over three months after the onset of heavy rains and no malaria epidemic was recorded with weekly malaria incidence consistently below 0.5 per 1000 population per week (100 times less than in 1998).

The big question is whether these vector control interventions triggered by environmental cues, prevented a malaria epidemic? This is difficult to definitively answer, but the neighboring county of Greater Garissa County provides some sort of control arm. This county has similar characteristics to Wajir (semi arid) and experienced very similar atypical environmental conditions as Wajir County in 2006/2007 with massive rainfall followed by flooding, mass population displacement, and human/animal cases of RVF [16]. Malnutrition rates during the same time period were similar to those experienced in Wajir, reaching a mean of 24% global acute malnutrition in 2006 [17]. In this case, donors were slow to respond and large scale vector control interventions by the MENTOR initiative in Garissa did not start until March 2007, over six months after the start of heavy rains, which is similar to what happened in Wajir in 1997/1998. In Garissa County, there was almost a doubling of reported malaria cases from 7719 in 2006 to 13739 in 2007, and monthly malaria incidence peaked at 42 per 1000 population in January 2007 and continued at high levels of above 30 per 1000 per month for the next three to four months [16]. Thus, while impossible to formally compare these two regions due to differences in data collection systems, Garissa can be said to have experienced similar climatic events as Wajir, malaria control interventions were started much later, and there was a significant increase in malaria cases [16].

There are several strengths to this study. Weekly malaria incidence figures at the town level in Wajir were based on clinical features observed first-hand by MSF combined with thin blood smears showing malaria parasites or positive malaria rapid diagnostic tests. There was also a robust multidisciplinary approach with several different and concurring sources of data on the measurement of environmental changes and the impact on the population such as rates of malnutrition and RVF.

Limitations relate to the operational nature of the study. First, malaria admission data from Wajir hospital during the two time periods were obtained from different sources, they did not describe the same populations and the diagnosis might have been made without parasite or serological confirmation. Second, the catchment area for Wajir Hospital might have been the county as well as the town, and it was principally the town that was targeted for vector control interventions by MSF. Third, we only have second-hand data over a limited period of time, from a control county such as Garissa, collected through separate channels precluding formal comparison - more data would have helped to strengthen the case for linking timely vector control interventions and malaria prevention in Wajir county in 2006/2007. Additionally, there was a report by Epicentre that in the neighboring county of Ijara, no malaria outbreak occurred between 22 January and 11 February 2007. While distribution of insecticide-treated bed nets reportedly also took place in this county, the scope and timing of such interventions could not be discerned, and it is therefore difficult to assess whether Ijara can be regarded as a control county for Wajir [18].

The rainfall was truly exceptional in 1996–1998. In 2006 there was massive rainfall, although not to the level or the duration experienced ten years previously. However, major outbreaks of RVF have occurred in this county only during the two study periods. This phenomenon requires heavy rainfall and extensive flooding of low lying grassland depressions associated with the rapid and mass emergence of *Aedes* mosquitoes, and such episodes of RVF often herald malaria epidemics in this sort of context. Furthermore, in Wajir county the high temperatures which stayed above 22°C favor the development of the malaria parasite in the Anopheles mosquitoes [19].

Wajir County exemplifies the epidemiology of an unstable malaria zone: this is typified by limited overall rainfall, protracted periods of drought with few infective bites, limited transmission of *Plasmodium falciparum* and thus low collective immunity of the population for malaria which is then followed by heavy rainfall leading to conditions that are ripe for a true malaria epidemic. Other people living in moderate to high malaria transmission regions of the country experience many infective bites throughout the year, and the children that survive repeated malaria infections in their first five years of life develop partial protective immunity to severe *P. falciparum* malaria [5], [19], [20], [21]. Periods of drought also cause widespread malnutrition and there is evidence that malaria prevalence is further increased in such circumstances [22].

Vector control interventions, such as the ones used in the study, are well-established methods for controlling malaria at the community level [23], [24], [25]. However, in an area with seasonal malaria transmission it seems vital to implement these interventions in a timely way. For this, there needs to be sufficient information for Ministries of Health and Aid agencies to raise a red early warning flag to prevent a malaria epidemic from occurring [26]. This study suggests that very heavy rainfall in an area like Wajir County that is followed by flooding and reports of RVF should be enough to trigger a wide scale vector control intervention to prevent epidemic malaria. This concurs with previous suggestions [2], [27] that area specific monitoring of rainfall is very important, and that this can provide key early epidemic warning indicators to inform responsible stakeholders of the need to mount rapid integrated epidemic prevention responses.

In conclusion, this study suggests that atypical environmental conditions with massive rainfall, flooding and RVF following a period of drought can herald a malaria outbreak in semi arid settings such as the North Eastern Province of Kenya, and that this could be prevented by timely, preemptive and rapid delivery of appropriate vector control interventions. A paradigm shift might be needed that allows resources to be made available to start rapid and widespread scale up of vector control interventions before any significant rise in malaria cases is declared.
